# Temporal Trends and Clinical Impact of Malnutrition on In-Hospital Outcomes Among Patients with Advanced Chronic Kidney Disease: A Nationwide Inpatient Analysis

**DOI:** 10.3390/nu17091508

**Published:** 2025-04-29

**Authors:** Wannasit Wathanavasin, Charat Thongprayoon, Wisit Kaewput, Supawit Tangpanithandee, Supawadee Suppadungsuk, Wisit Cheungpasitporn

**Affiliations:** 1 Division of Nephrology and Hypertension, Department of Medicine, Mayo Clinic, Rochester, MN 55905, USA; thongprayoon.charat@mayo.edu (C.T.); supawit_d@hotmail.com (S.T.); s.suppadungsuk@hotmail.com (S.S.); 2 Nephrology Unit, Department of Medicine, Charoenkrung Pracharak Hospital, Bangkok Metropolitan Administration, Bangkok 10120, Thailand; 3 Department of Military and Community Medicine, Phramongkutklao College of Medicine, Bangkok 10400, Thailand; wisitnephro@gmail.com; 4 Chakri Naruebodindra Medical Institute, Faculty of Medicine Ramathibodi Hospital, Mahidol University, Samut Prakan 10540, Thailand

**Keywords:** malnutrition, chronic kidney disease, prevalence, mortality, in-hospital outcomes, nationwide study

## Abstract

Background/Objectives: Malnutrition is a prevalent yet under-recognized condition in patients with advanced chronic kidney disease (CKD), contributing to increased morbidity, mortality, and healthcare burden. The aim of this study is to determine the prevalence and trends of malnutrition and investigate the impact of malnutrition on in-hospital outcomes, treatments, and resource utilization in hospitalized patients with advanced CKD. Methods: This study utilized the National Inpatient Sample (NIS) database to identify hospitalized patients with advanced CKD from 2016 to 2021. This study investigated temporal trends in the prevalence and in-hospital mortality across different degrees of malnutrition in advanced CKD patients. Multivariable regression models were used to assess the association between malnutrition and in-hospital outcomes. Results: Out of 1,244,415 advanced CKD patients, 67,587 (5.4%) had mild to moderate malnutrition, and 63,785 (5.1%) had severe malnutrition. Malnourished patients exhibited significantly higher in-hospital mortality, with adjusted odds ratios of 1.70 (95% confidence interval (CI), 1.64–1.75) for mild to moderate cases and 2.67 (95% CI, 2.60–2.75) for severe cases. Severely malnourished patients were associated with longer mean hospital stay by 7.0 days and higher hospitalization costs by $97,767 compared with non-malnourished patients. The prevalence of severe malnutrition showed a significant uptrend from 4.2% in 2016 to 5.5% in 2021 (*p* for trend < 0.001). Conclusions: Malnutrition in advanced CKD is an increasingly prevalent condition linked to worsened in-hospital outcomes and heightened healthcare resource utilization. The rising trend of severe malnutrition underscores the need for early nutritional screening and the need for future interventional studies to mitigate adverse clinical outcomes in this high-risk population.

## 1. Introduction

On a global scale, chronic kidney disease (CKD) ranks as the third fastest-growing cause of death and stands out as the only non-communicable disease (NCD) with a sustained increase in age-adjusted mortality [1]. By 2040, CKD is expected to be the fifth highest cause of years of life lost [2]. Moreover, as a comorbidity, CKD is a significant predictor of hospital admissions, with hospitalization rates more than twice as high in individuals with CKD compared to those without CKD [3]. Hospitalizations in this population, especially for those with end-stage kidney disease (ESKD), are more likely to result in in-hospital complications, contributing to longer hospital stays and higher healthcare costs [4].

Malnutrition is one of the crucial issues that is prevalent but often under-recognized and undertreated in hospitalized ESKD patients [5]. The American Society of Parenteral and Enteral Nutrition defines malnutrition as an acute, subacute, or chronic state of nutrition in which varying degrees of overnutrition or undernutrition, with or without inflammation, cause changes in body composition and impaired function [6]. According to the World Health Organization (WHO), malnutrition is characterized by either a deficiency or excess of nutrient intake, an imbalance in essential nutrients, or the impaired utilization of nutrients. Undernutrition, specifically, is commonly manifested in four main forms: wasting, stunting, underweight, and micronutrient deficiencies [7]. The majority of ESKD patients suffer from protein–calorie malnutrition, which results from the complex pathophysiology associated with impaired kidney function. These include various metabolic and endocrine abnormalities, such as decreased levels of erythropoietin, vitamin D, carnitine, testosterone, and thyroid hormones, as well as insulin resistance [8]. In addition, the accumulation of uremic toxins contributes to appetite suppression, leading to reduced food intake and insufficient fulfillment of a patient’s physiological requirements [9]. Furthermore, dialysis-induced hypercatabolism increases amino acid losses, exacerbating negative nitrogen balance, particularly on dialysis days [10]. Ultimately, if these processes persist and remain untreated, nutrient stores become depleted, leading to the final clinical phase of malnutrition, characterized by a significant loss of a patient’s organ and physical function [11,12,13].

Although several studies [14,15,16] have highlighted the detrimental effects of malnutrition in CKD patients, only a few have addressed its association with in-hospital outcomes. A study by Ruiz et al. [17] demonstrated that a significant number of patients were malnourished upon hospital admission, which was linked to prolonged hospital stays, increased mortality, and greater overall hospitalization expenses. In addition, Starke et al. [18] found that addressing malnutrition in hospitalized patients improved both their nutritional status and quality of life, resulting in fewer complications, decreased antibiotic requirements, and reduced re-hospitalizations. However, it is worth noting that these studies did not focus specifically on patients with CKD. Therefore, our study aims to determine the prevalence trends of malnutrition and investigate whether malnutrition contributes to higher in-hospital mortality, adverse clinical outcomes, inpatient treatments, and healthcare resource utilization in patients with stage 5 CKD, including those who are non-dialysis dependent (5ND)—meaning their kidney function is severely reduced (eGFR < 15 mL/min/1.73 m^2^) but they have not yet started dialysis due to the absence of clinical indications—and those receiving maintenance dialysis (5D), including hemodialysis and peritoneal dialysis, once clinical indications are met. Understanding the trends in malnutrition prevalence and its association with in-hospital outcomes would highlight the importance of nutritional status, prioritize nutritional care, and ultimately help to alleviate the future disease burden and economic costs of hospitalized patients with advanced CKD.

## 2. Materials and Methods

### 2.1. Data Sources and Patient Sample

The National Inpatient Sample (NIS) is the largest publicly accessible all-payer inpatient care database in the United States. This database contains data on over 7 million hospital admissions and a weighted estimate of 35 million annual hospital stays, derived from a 20% stratified sample of over 4000 hospitals. The NIS provides data on demographic details, socioeconomic status, diagnoses, comorbidities, length of stay, and total hospital cost for each hospitalization. These charges reflect the full amount billed by hospitals. Detailed information is available on the official website at https://www.hcup-us.ahrq.gov (accessed on 15 January 2025). Because the database does not include identifiable patient information, institutional review board approval is not required.

### 2.2. Study Population

This study utilized the NIS database from 2016 to 2021 and included adult patients (aged ≥ 18 years) with advanced CKD. The patients were identified using the International Classification of Diseases, Tenth Revision, Clinical Modification (ICD-10-CM) coding system. We employed the ICD-10-CM codes (refer to Supplementary Table S1) to select all hospitalized patients with stage 5 chronic kidney disease stage (CKD), including both non-dialysis (5ND) and receiving dialysis (5D)—specifically those on hemodialysis (HD) and peritoneal dialysis (PD)—from the NIS data between 2016 and 2021. Patients with acute kidney injury (AKI), kidney transplants, or CKD stages 1–4 were excluded. Malnutrition was identified using ICD-10 codes and classified into two categories depending on severity: (1) mild to moderate malnutrition (E44.0, E44.1, E46) and (2) severe malnutrition (E40, E41, E42, E43, R64). The use of these codes to describe the severity of malnutrition was defined previously in the literature [19]. A full list of the ICD-10 codes used in this study is provided in Supplementary Table S1.

### 2.3. Covariates and Outcomes Measure

The study variables included age at the time of admission, sex, race (White, Black, Hispanic, and Asian or Pacific Islander), dialysis status, and comorbidity measures. These included the Charlson Comorbidity Index—which encompasses 19 chronic conditions and is commonly used to predict one-year mortality, particularly in time-limited clinical settings due to its simplicity—and the Elixhauser Comorbidity Index, which includes 30 (or in some versions, 31) comorbidities and is used to predict a broader range of in-hospital outcomes, including in-hospital mortality, length of stay, adverse events, and hospital discharges. Additional comorbidities considered were diabetes mellitus, hypertension, dyslipidemia, congestive heart failure, coronary artery disease, cerebrovascular disease, peripheral vascular disease, cirrhosis, cancer, and dementia or cognitive impairment. Other variables included smoking history, alcohol use, admission type (elective or non-elective), hospital length of stay, hospitalization cost, and hospital location and teaching status. The primary outcomes of this study were the prevalence of malnutrition and its association with in-hospital mortality in patients with CKD stages 5ND and 5D. The secondary outcomes were categorized into three groups: (1) adverse clinical outcomes (sepsis, catheter-related bloodstream infections (CRBSIs), and volume overload), (2) inpatient treatments (use of vasopressors, total parenteral nutrition (TPN), mechanical ventilator and blood transfusions), and (3) resource utilization (length of hospital stay and hospitalization costs).

### 2.4. Statistical Analysis

Clinical characteristics, outcomes, and resource utilization between mild to moderate malnutrition, severe malnutrition, and no malnutrition were compared using one-way ANOVA for continuous variables and the Chi-squared test for categorical variables. Categorical variables are presented as percentages. Continuous variables with a normal distribution are reported as mean ± standard deviation (SD), while skewed continuous variables are expressed as median with interquartile ranges (IQRs). The annual inpatient prevalence of malnutrition among advanced CKD patients was calculated by dividing the number of hospitalized advanced CKD patients with malnutrition, categorized by severity, by the total weighted hospitalized advanced CKD population for each year from 2016 to 2021. Temporal trend analysis was conducted to identify significant changes in the prevalence of malnutrition and the in-hospital mortality rate over time. The analysis included a visual inspection for trends and the calculation of annual percentage changes among advanced CKD patients. Comparative analysis was performed across different levels of malnutrition severity to assess temporal trends, using the Cochran–Armitage trend test for prevalence and the Jonckheere–Terpstra test for in-hospital mortality.

The associations between malnutrition, adverse clinical outcomes, and inpatient treatments were assessed using logistic regression analysis, while the association between malnutrition and resource utilization was assessed using linear regression analysis. The association was adjusted for potential patient-level factors, including age, gender, race, mode of kidney replacement therapy (KRT), smoking, alcohol use status, admission type, comorbid conditions, and hospital-level factors, including location and teaching status, in the multivariable analysis. Odds ratios (Ors) with 95% confidence intervals (Cis) were reported for binary outcomes, and mean differences with 95% CIs were reported for the numerical outcomes. A two-tailed *p*-value < 0.05 was considered statistically significant. All analyses were performed using Stata, version 16 (StataCorp LLC, College Station, TX, USA).

## 3. Results

### 3.1. Patient Characteristics

A total of 1,244,415 advanced CKD patients were identified from the NIS database between 2016 and 2021. Of these, 131,372 (10.6%) patients had malnutrition. Mild to moderate malnutrition was observed in 67,587 (5.4%) patients, whereas severe malnutrition was present in 63,785 (5.1%) patients. Table 1 provides a comparison of the demographic and hospital-related characteristics of the advanced CKD patients with and without malnutrition. Compared to the advanced CKD patients without malnutrition, those with malnutrition were more likely to be older, White, and have a history of alcohol use, along with a higher prevalence of chronic conditions, resulting in a higher Charlson Comorbidity and Elixhauser Comorbidity index. Detailed information on the comorbidities revealed that the patients with malnutrition had a higher proportion of congestive heart failure, cerebrovascular disease, peripheral vascular disease, cirrhosis, malignancy, and dementia/cognitive impairment. In terms of resource utilization, the patients with malnutrition had a longer length of stay and higher hospitalization costs.

### 3.2. Prevalence of Malnutrition in Hospitalized Advanced CKD Patients, Stratified by Dialysis Status

Out of the 1,244,415 hospitalized advanced CKD patients, 131,372 were diagnosed with malnutrition, resulting in an overall prevalence rate of 10.6%. In terms of dialysis status, malnutrition was most prevalent among the PD patients (11.6%), followed by the HD patients (11.1%), and least common in the non-dialysis population (9.9%) (Table 2).

### 3.3. Trends in Mild to Moderate/Severe Malnutrition Prevalence and In-Hospital Mortality

**Figure 1:**
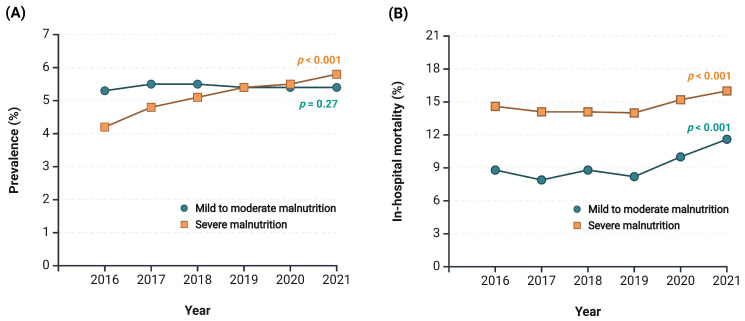
Temporal trends in malnutrition prevalence (**A**) and in-hospital mortality (**B**) in advanced CKD patients with different levels of malnutrition severity.

The prevalence of severe malnutrition in hospitalized patients with advanced CKD significantly increased from 4.2 in 2016 to 5.5% in 2021 (*p* for trend test < 0.001; Figure 1A), whereas the prevalence of mild to moderate malnutrition did not change significantly (*p* for trend = 0.27). In-hospital mortality associated with malnutrition significantly increased from 8.8% to 11.6% for mild to moderate malnutrition (*p* for trend < 0.001) and from 14.6% to 15.2% for severe malnutrition (*p* for trend test < 0.001; Figure 1B).

### 3.4. Association Between Mild to Moderate/Severe Malnutrition and In-Hospital Mortality

**Figure 2:**
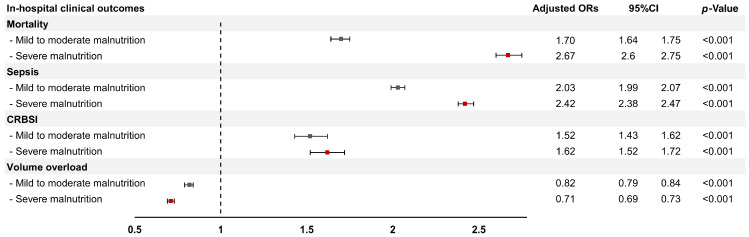
Forest plot illustrating the adjusted odds ratios (and 95% confidence intervals) for the association between malnutrition and in-hospital clinical outcomes, categorized by severity of malnutrition (mild to moderate/severe) among hospitalized advanced CKD patients. CI, confidence interval; ORs, odds ratios; CRBSI, catheter-related bloodstream infection.

The overall in-hospital mortality rate for advanced CKD patients was 5.5%. The in-hospital mortality rate was higher in the severe malnutrition group (14.7%) and the mild to moderate malnutrition group (9.2%) compared with the non-malnutrition group (4.6%) (*p* < 0.001). After adjusting for demographic factors, comorbidities, and hospital-level factors, a multivariable logistic regression analysis demonstrated that mild to moderate malnutrition was significantly associated with an increased risk of in-hospital mortality (adjusted OR 1.70, 95% CI 1.64–1.75), while severe malnutrition was associated with a substantially higher risk of mortality (adjusted OR 2.67, 95% CI 2.60–2.75) (Figure 2 and Table 3).

### 3.5. Association Between Mild to Moderate/Severe Malnutrition and Adverse Clinical Outcomes

In the multivariable logistic regression analysis (Figure 2), mild to moderate malnutrition was significantly associated with a higher risk of sepsis (adjusted OR 2.03, 95% CI, 1.99–2.07) and CRBSI (adjusted OR 1.52, 95% CI 1.43–1.62), but it was associated with a lower risk of volume overload (adjusted OR 0.82, 95% CI 0.79–0.84) among hospitalized patients with advanced CKD. Similarly, severe malnutrition was associated with increased risks of sepsis (adjusted OR 2.42, 95% CI 2.38–2.47) and CRBSI (adjusted OR 1.62, 95% CI 1.52–1.72), but it was associated with a lower risk of volume overload (adjusted OR 0.71, 95% CI 0.69–0.73), as presented in Table 3.

### 3.6. Association Between Mild to Moderate/Severe Malnutrition and Inpatient Treatments

In terms of inpatient treatments, the advanced CKD patients with mild to moderate malnutrition were at an increased risk of requiring vasopressor (adjusted OR 1.89, 95% CI 1.79–2.00), TPN (adjusted OR: 7.78, 95% CI 7.12–8.50), mechanical ventilation (adjusted OR 1.11, 95% CI 1.06–1.15), and blood transfusions (adjusted OR: 1.61, 95% CI 1.57–1.66). Likewise, the ESKD patients with severe malnutrition had a higher risk of requiring vasopressors (adjusted OR 2.32, 95% CI 2.21–2.44), TPN (adjusted OR 11.94, 95% CI 10.99–12.97), mechanical ventilation (adjusted OR 1.19, 95% CI 1.15–1.24), and blood transfusions (adjusted OR 1.77, 95% CI 1.72–1.82) compared to the advanced CKD patients without malnutrition (Table 3).

### 3.7. Association Between Mild to Moderate/Severe Malnutrition and Resource Utilization

Regarding resource utilization, the advanced CKD patients with mild to moderate malnutrition had a longer length of hospital stay by 5.3 days (95% CI 5.2–5.5) and higher hospitalization costs by $ 73,130 (95%CI 69,593–76,666) compared to the advanced CKD patients without malnutrition. Similarly, those with severe malnutrition had a longer hospital stay by 7.0 days (95% CI 6.8–7.2) and higher hospitalization costs by $97,767 (95% CI 92,805–102,728) than the advanced CKD patients without malnutrition (Table 3).

## 4. Discussion

**Figure 3:**
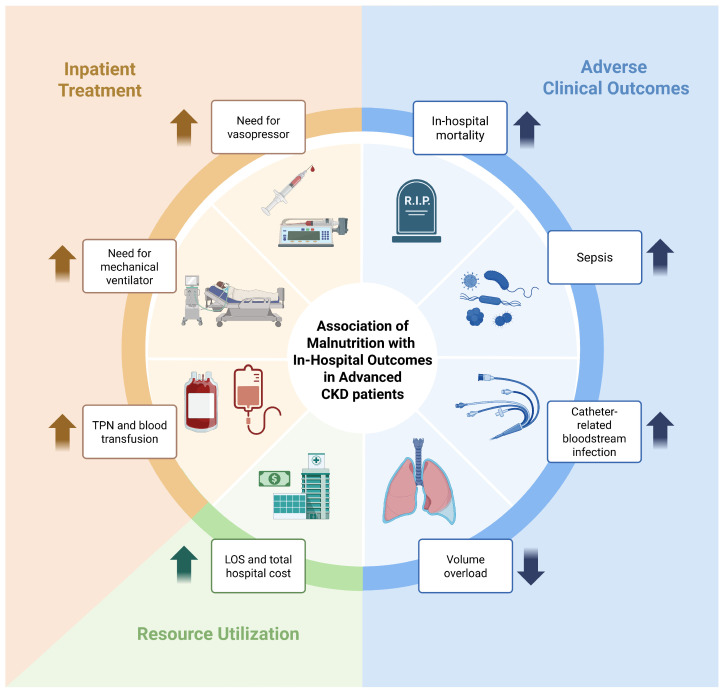
Summary of the association between malnutrition and adverse clinical outcomes, inpatient treatments, and resource utilization among hospitalized advanced CKD patients. Abbreviations: CKD, chronic kidney disease; LOS, length of stay; TPN, total parenteral nutrition. This picture was created with BioRender.com.

To the best of our knowledge, this is the first study to report the prevalence trends and their associations with in-hospital outcomes, including mortality, adverse clinical outcomes, inpatient treatments, and resource utilization, among malnourished patients with CKD stages 5ND and 5D. Our analysis highlighted a notable increase in the prevalence of severe malnutrition among hospitalized advanced CKD patients, from 4.2% in 2016 to 5.5% in 2021. This trend also aligns with an increase in in-hospital mortality rates, from 8.8% to 11.6% for mild to moderate cases and from 14.6% to 15.2% for severe cases. Additionally, malnutrition was associated with an increased risk of in-hospital mortality, sepsis, and CRBSI, albeit a lower risk of volume overload, regardless of its severity. Malnourished inpatients with advanced CKD required more intensive inpatient care, including vasopressors, TPN, mechanical ventilation, and blood transfusions, leading to longer hospital stays and higher overall costs compared to non-malnourished individuals (Figure 3).

Disease-related malnutrition is prevalent among patients with CKD and significantly impacts both morbidity and mortality [20]. As the number of CKD diagnoses continues to rise, malnutrition presents an increasingly urgent clinical challenge [21]. In our nationwide study of more than 1 million hospitalized advanced CKD patients, we observed a significant increase in the prevalence trends of malnutrition, particularly in its severe form. It is evident that malnutrition is more frequently observed in dialysis than in pre-dialysis patients (Table 2). This finding can be primarily attributed to the dialytic procedure, which induces a net protein catabolic state, influenced by the dialysis technique used and a systematic inflammatory response related to the biocompatibility of the dialysis system. As a result, this contributes to amino acid/protein loss and increased energy expenditure during dialysis [22]. Moreover, recent studies indicate that overzealous dietary and fluid restrictions in the dialysis population may inherently lead to lower protein and calorie intake, exacerbating the effects of uremia-induced anorexia [23,24]. Improving overall health requires prioritizing more liberalized diets, along with a greater intake of a variety of nutritious foods [25,26]. Based on our study, malnutrition is slightly more prevalent among PD patients compared to HD patients, although both modalities carry a high risk for malnutrition, as previously noted. Similarly, Kim et al. [27] pointed out that PD patients had poorer dietary behaviors and subsequently less sufficient nutrition intake compared to those of HD patients. However, their study is limited by a small sample size, a cross-sectional design, and its reliance on dietary behavioral surveys and a Semi-Quantitative Food Frequency Questionnaire (Semi-FFQ) for nutritional assessment. Another important issue may be related to the frequency of hospitalization among this population. In a study by Sluijs et al. [28], which followed 695 dialysis patients, those receiving PD had higher hospitalization rates—mainly due to peritonitis—as well as increased risks for first-time admissions, more total hospitalizations, and longer hospital stays per year compared to patients on HD. It is well recognized that during hospital stays, patients may develop iatrogenic malnutrition, also referred to as “physician-induced malnutrition,” which can result from medical interventions, pharmacological therapies, prolonged hospitalization, or hospital-acquired infections. On a larger scale, a global meta-analysis involving 16,434 patients from 10 geographical regions found no statistically significant difference in the prevalence of CKD-related malnutrition or protein energy wasting (PEW) between HD and PD patients, with HD patients showing prevalence rates of 28–56% (median 43%) and PD patients showing rates of 32–39% (median 36%), with a *p* for interaction of 0.915 [29]. Therefore, this issue remains inconclusive and warrants further investigation.

In ESKD patients, maintaining proper nutritional status is essential, as malnutrition can cause various adverse clinical consequences and may indicate the presence of inflammation and immune dysfunction [30]. We discovered that even after adjusting for demographic factors, comorbidities, and hospital-level variables, malnutrition was still linked to an increased risk of in-hospital mortality (adjusted OR of 1.70 for mild to moderate malnutrition and 2.67 for severe malnutrition, with *p*-value < 0.001). There are multiple underlying mechanisms through which malnutrition influences survival outcomes, as demonstrated in our study. One key factor is that malnutrition compromises immune function, increasing individuals’ vulnerability to infections [31]. In our nationwide study, we found that malnutrition, at any severity level, was significantly associated with an increased risk of sepsis and CRBSI in hospitalized advanced CKD patients (Table 3). Sepsis and bacterial infections related to CRBSI are very common among ESKD patients, with sepsis emerging as the second leading cause of death, following cardiovascular disease [32,33]. In patients with impaired kidney function, a dysregulated host response has been described, characterized by defective phagocytic function of granulocytes, impaired monocyte function, and compromised T lymphocyte maturation [34,35,36]. Our findings align with a clinical study by Bou et al. [37], which demonstrated that ESKD is associated with higher odds (OR 1.44, 95% CI 1.03–1.53) of hospital mortality in septic patients admitted to the intensive care unit compared to non-ESKD individuals. Moreover, the immunocompromised state of uremia, aging, and diabetes, along with the frequent use of intravascular catheters in HD patients, have been linked to higher rates of infection [38]. Ultimately, the double trouble of diminished kidney function and undernutrition weakens immune function, placing patients at a very high risk for infectious complications and increased mortality [39].

While malnutrition is linked to worsening various adverse clinical outcomes, we found it intriguingly associated with a lower risk of volume overload (Table 3). Since restricting sodium and fluid intake is a standard recommendation to prevent volume overload in advanced CKD patients, this may partially explain our finding, where malnutrition is associated with decreased nutritional intake, which also leads to reduced fluid and sodium intake, thereby decreasing the risk of fluid accumulation. However, malnutrition in CKD is not solely attributed to reduced nutritional intake. Several experimental and clinical studies [40,41,42] have shown that tissue sodium accumulation and volume overload share a similar pathophysiological pathway with malnutrition/PEW via insulin resistance and inflammation. In addition, hypoalbuminemia, related to malnutrition and/or inflammation during hospitalization [43], may also lead to fluid redistribution to the interstitial compartment, hindering fluid removal during dialysis therapy in these patients [44,45]. This suggests possible unmeasured confounding factors that could not be completely accounted for given the constraints of our data, including residual kidney function in the dialysis population, dialysis frequency and prescriptions, as well as nutritional intervention—all of which could influence patients’ fluid balance. Although the interplay between these conditions is complex and not fully understood, they should be addressed simultaneously and without delay in clinical practice. Additionally, the paradoxical association between higher CHF prevalence and lower volume overload in malnourished patients may reflect diagnostic overshadowing or coding artifacts, where volume overload is subsumed under the CHF diagnosis and not separately coded. Despite adjusting for CHF in multivariable models, residual confounding from coding practices cannot be fully excluded.

The detrimental effects of malnutrition are not limited to poor clinical outcomes but also affect inpatient treatments and resource utilization. As shown in the baseline characteristics in Table 1, malnourished patients tended to be older, had a higher prevalence of comorbidities (as defined by the Elixhauser Comorbidity Index), and were less likely to be admitted electively. These factors can influence inpatient treatments and resource utilization beyond the effects of malnutrition. To account for this, we performed a multivariable analysis adjusting for these factors, yet it still demonstrated a higher intensity care of inpatient treatments and resource utilization. Usually, for several reasons, malnourished patients are at higher risk of sepsis, as previously mentioned, which may further require vasopressors and mechanical ventilation. Sepsis in this population is frequently more severe, progressing to septic shock, which results in critical illness and demands more intensive care [37]. Moreover, our study observed a significant increase in the use of nutritional intervention, particularly total parenteral nutrition, to meet the patients’ nutritional requirement, with adjusted OR values of 7.78 for mild to moderate malnourished and 11.94 for severe malnourished patients with advanced CKD (Table 3). According to the ASPEN guidelines [46], parenteral nutrition (PN) also plays a crucial role in malnourished patients who have contraindications to enteral nutrition (EN), cannot tolerate adequate EN, or lack of bowel function, in order to restore their nutritional status. Although PN might serve as an effective bridge, enabling patients to recover sufficiently to undergo specific treatments or surgical interventions, it is a costly therapy, especially in its customized form [47]. In CKD patients, anemia is common, with its severity increasing as the disease progresses. Besides erythropoietin deficiency, anemia due to nutritional deficiencies, including iron, folate, and vitamin B12, is a major contributor to CKD-related anemia [48]. Moreover, in hospital settings, inflammation resulting from malnutrition often worsens anemia of inflammation. GI bleeding is a serious complication among hospitalized dialysis patients and is strongly linked to higher in-hospital mortality [49]. In their nationwide study, Tsai et al. [50] observed that admissions for lower GI bleeding were significantly more frequent in dialysis patients than in those with non-dialysis CKD or healthy controls (12.9%, 3.6%, and 2.8%, respectively; *p* < 0.001). Expanding on this, Huang et al. [49] found that patients on hemodialysis had a 1.13-fold greater risk of GI bleeding compared to those on peritoneal dialysis. This increased risk may be due to the more intensive use of anticoagulants during the HD procedure, particularly during hospitalization. Considering all these factors helps to explain our result that malnourished advanced CKD patients require blood transfusions 1.6–1.8 times more often than those without malnutrition (Table 3). As a result, more adverse in-hospital complications and inpatient treatments come up with more resource utilization, including longer length of stay and total hospitalization costs. These findings may highlight the need for comprehensive nutritional assessments and the implementation of nutrition strategies to improve nutritional status and lower economic healthcare costs for hospitalized CKD patients, particularly those in advanced stages.

The major strengths of our study lie in the use of a large, nationally representative U.S. database, which offered strong statistical power. In addition, this study presented trends in malnutrition prevalence categorized by severity level, while also covering its comprehensive association with in-hospital outcomes, including major adverse clinical outcomes, inpatient treatments, and resource utilization. We also made thorough adjustments for all potential confounding factors that could influence the outcomes of interest. However, this study still has several limitations. Firstly, this is a retrospective observational study using a registry database. Thus, the data in this report must be interpreted as associations rather than causal relationships. Moreover, our nationwide analysis, based on the U.S. healthcare system, included only a small proportion of Asian individuals (up to 5%), and healthcare costs and practices differ across regions. These factors limit the generalizability of our findings to the global population. Secondly, with regard to nutritional assessments, we cannot specify the tools used, nor can we ensure that all assessments were conducted by certified nutrition professionals, particularly registered dietitians. Malnutrition cases in this study were determined solely by ICD-10-CM codes recorded in hospital discharge records. Although validated codes have proven high accuracy and positive predictive value for malnutrition [51,52], potential undercoding (failure to detect conditions) or coding errors (incorrect categorization of conditions) are inevitable. As research suggests that malnutrition may be underdiagnosed, it is likely that some malnourished patients were not identified through coding and were categorized as non-malnourished. Therefore, the overall malnutrition prevalence in this 2016 to 2021 national sample likely represents only a fraction of the true prevalence of hospitalized advanced CKD patients. However, the trends in prevalence are also crucial for encouraging further attention to early detection and implementation of optimal nutritional strategies to alleviate the disease burden. Lastly, while important, medication use and laboratory parameters were not provided for patients in the NIS database and, therefore, could not be incorporated into the data synthesis. Future prospective studies are needed to address this gap.

## 5. Conclusions

In conclusion, this nationwide study confirmed that the prevalence of malnutrition, especially in its severe form, increased among hospitalized advanced CKD patients from 2016 to 2021, alongside higher in-hospital mortality. Additionally, the association between malnutrition and in-hospital outcomes was evident through higher adverse clinical outcomes (apart from volume overload), more intensive inpatient treatments, and increased resource utilization compared to those without malnutrition. The impact on these outcomes is proportional to the degree of malnutrition, with severe malnutrition associated with worse outcomes compared to mild to moderate malnutrition. Given these findings, early recognition and timely intervention for malnutrition are crucial to prevent or reverse disease progression. Future well-designed prospective studies should further investigate whether nutritional interventions can improve clinical outcomes and reduce healthcare economic resources in hospitalized advanced CKD patients with malnutrition.

## Figures and Tables

**Table 1:** Patient characteristics of advanced CKD patients by malnutrition status.

Variables	No Malnutrition (*n* = 1,113,043)	Malnutrition		*p*-Value
		Mild to Moderate (*n* = 67,587)	Severe (*n* = 63,785)	
Age (years)	61.3 ± 15.3	64.8 ± 14.8	65.4 ± 14.5	<0.001
Male sex, *n* (%)	608,460 (54.7)	35,537 (52.6)	35,174 (55.2)	<0.001
Race, *n* (%)				
White	440,474 (42.3)	28,666 (45.3)	27,388 (45.9)	<0.001
Black	369,407 (35.4)	21,207 (33.5)	20,476 (34.3)	
Hispanic	188,573 (18.1)	10,407 (16.4)	8797 (14.7)	
Asian or Pacific Islander	43,859 (4.2)	3040 (4.8)	3024 (5.1)	
Dialysis status, *n* (%)				
Non-dialysis	513,974 (46.2)	29,471 (43.6)	26,603 (41.7)	<0.001
Hemodialysis	556,788 (50.0)	34,986 (51.8)	34,764 (54.5)	
Peritoneal dialysis	42,281 (3.8)	3130 (4.6)	2418 (3.8)	
Charlson comorbidity score, median (IQR)	5 (4–6)	5 (4–7)	5 (4–7)	<0.001
Elixhauser score, median (IQR)	6 (4–7)	7 (6–8)	7 (6–8)	<0.001
Comorbidity, *n* (%)				
Diabetes mellitus	712,772 (64.0)	41,117 (60.8)	33,470 (52.5)	<0.001
Hypertension	1,057,154 (95.0)	62,828 (93.0)	57,409 (90.0)	<0.001
Dyslipidemia	455,298 (40.9)	24,768 (36.7)	20,445 (32.1)	<0.001
Congestive heart failure	536,736 (48.2)	35,531 (52.6)	32,598 (51.1)	<0.001
Coronary artery disease	178,875 (16.1)	10,594 (15.7)	9422 (14.8)	<0.001
Cerebrovascular disease	94,509 (8.5)	7726 (11.4)	6978 (10.9)	<0.001
Peripheral vascular disease	148,460 (13.3)	10,883 (16.1)	9847 (15.4)	<0.001
Cirrhosis	95,264 (8.6)	9350 (13.8)	10,972 (17.2)	<0.001
Malignancy	51,170 (4.6)	5089 (7.5)	6962 (10.9)	<0.001
Dementia/cognitive impairment	59,434 (5.3)	6493 (9.6)	7062 (11.1)	<0.001
Smoking, *n* (%)	250,756 (22.5)	12,232 (18.1)	11,078 (17.4)	<0.001
Alcohol use, *n* (%)	25,986 (2.3)	2643(3.9)	3227 (5.1)	<0.001
Elective admission, *n* (%)	99,349 (8.9)	5066 (7.5)	3691 (5.8)	<0.001
Length of stay, days, median (IQR)	4 (2–8)	8 (4–15)	8 (4–16)	<0.001
Hospitalization cost ($), median (IQR)	48,155 (25,571–96,002)	80,674 (40,733–173,328)	84,957 (41,558–186,404)	<0.001
Hospital location/teaching status, *n* (%)				
Rural	55,734 (5.0)	2745 (4.1)	2706 (4.2)	<0.001
Urban—nonteaching	251,592 (19.4)	13,612 (20.1)	12,077 (18.9)	
Urban—teaching	841,717 (75.6)	51,230 (75.8)	49,002 (76.8)	

**Table 2:** Prevalence of malnutrition in hospitalized patients with CKD stage 5, stratified by dialysis status.

Nutritional Status	Total, *n* (%) (*n* = 1,244,415)	Dialysis Status, *n* (%)			*p*-Value
		Non-Dialysis (*n* = 570,048)	HD (*n* = 626,538)	PD (*n* = 47,829)	
No malnutrition	1,113,043 (89.4)	513,974 (90.1)	556,788 (88.9)	42,281 (88.4)	<0.001
Mild to moderate malnutrition	67,587 (5.4)	29,471 (5.2)	34,986 (5.6)	3130 (6.5)	<0.001
Severe malnutrition	63,785 (5.1)	26,603 (4.7)	34,764 (5.5)	2418 (5.1)	<0.001

Abbreviations: HD, hemodialysis; PD, peritoneal dialysis.

**Table 3:** The association between severity of malnutrition (vs. no malnutrition) and in-hospital mortality, adverse clinical outcomes, and resource utilization in advanced CKD patients.

In-Hospital Outcomes	No Malnutrition (*n* = 1,113,043)	Mild to Moderate Malnutrition (*n* = 67,587)				Severe Malnutrition (*n* = 63,785)			
		Univariable Analysis		Multivariable Analysis		Univariable Analysis		Multivariable Analysis	
		OR (95% CI)	*p*-Value	Adjusted OR * (95% CI)	*p*-Value	OR (95% CI)	*p*-Value	Adjusted OR * (95% CI)	*p*-Value
**Primary outcome**									
In-hospital mortality	Ref.	2.07 (2.00–2.13)	<0.001	1.70 (1.64–1.75)	<0.001	3.51 (3.42–3.60)	<0.001	2.67 (2.60–2.75)	<0.001
**Secondary outcomes**									
**Adverse clinical outcomes**									
Sepsis	Ref.	2.24 (2.20–2.28)	<0.001	2.03 (1.99–2.07)	<0.001	2.79 (2.74–2.84)	<0.001	2.42 (2.38–2.47)	<0.001
Catheter-related bloodstream infection	Ref.	1.53 (1.44–1.63)	<0.001	1.52 (1.43–1.62)	<0.001	1.74 (1.64–1.85)	<0.001	1.62 (1.52–1.72)	<0.001
Volume overload	Ref.	0.74 (0.72–0.77)	<0.001	0.82 (0.79–0.84)	<0.001	0.64 (0.62–0.66)	<0.001	0.71 (0.69–0.73)	<0.001
**Inpatient treatments**									
Need for vasopressors	Ref.	2.30 (2.17–2.42)	<0.001	1.89 (1.79–2.00)	<0.001	3.12 (2.98–3.27)	<0.001	2.32 (2.21–2.44)	<0.001
TPN use	Ref.	8.40 (7.71–9.14)	<0.001	7.78 (7.12–8.50)	<0.001	13.95 (12.93–15.05)	<0.001	11.94 (10.99–12.97)	<0.001
Mechanical ventilation	Ref.	1.18 (1.14–1.23)	<0.001	1.11 (1.06–1.15)	<0.001	1.30 (1.25–1.35)	<0.001	1.19 (1.15–1.24)	<0.001
Blood transfusion	Ref.	1.75 (1.70–1.80)	<0.001	1.61 (1.57–1.66)	<0.001	2.03 (1.98–2.09)	<0.001	1.77 (1.72–1.82)	<0.001
**Resource utilization**									
Length of hospital stay (days)	Ref.	5.78 (5.12–5.95)	<0.001	5.32 (5.16–5.47)	<0.001	7.65 (7.44–7.86)	<0.001	7.00 (6.80–7.20)	<0.001
Hospitalization cost ($)	Ref.	79,019.5 (75,196.2–82,842.7)	<0.001	73,129.5 (69,592.8–76,666.2)	<0.001	107,529.6 (102,192.9–112,866.3)	<0.001	97,766.6 (92,805.4–102,727.8)	<0.001

* Adjusted for age, sex, race, year of hospitalization, Charlson comorbidity score, diabetes mellitus, hypertension, congestive heart failure, coronary artery disease, cerebrovascular disease, peripheral vascular disease, cirrhosis, cancer, dementia/cognitive impairment, smoking, alcohol drinking, hospital location/teaching status, mode of KRT, and admission type.

## Data Availability

The data underlying this article will be shared upon a reasonable request by the corresponding author due to privacy restriction.

## Supplementary Materials

The following supporting information can be downloaded at https://www.mdpi.com/article/10.3390/nu17091508/s1, Table S1: Diagnoses/procedures of interest and corresponding ICD-10 codes.

## Abbreviations

The following abbreviations are used in this manuscript:

AKI	acute kidney injury
CKD	chronic kidney disease
CRBSI	catheter-related bloodstream infection
EN	enteral nutrition
ESKD	end-stage kidney disease
HD	hemodialysis
ICD-10-CM	International Classification of Diseases, Tenth Revision, Clinical Modification
IQR	interquartile range
KRT	kidney replacement therapy
LOS	length of stay
NCD	non-communicable disease
NIS	National Inpatient Sample
ORs	odds ratios
PN	parenteral nutrition
PD	peritoneal dialysis
PEW	protein energy wasting
Semi-FFQ	Semi-Quantitative Food Frequency Questionnaire
SD	standard deviation
TPN	total parenteral nutrition
