# Supplementary material for: Temporal Trends and Clinical Impact of Malnutrition on In-Hospital Outcomes Among Patients with Advanced Chronic Kidney Disease: A Nationwide Inpatient Analysis
Source: Nutrients. 2025 Apr 29;17(9):1508. doi: 10.3390/nu17091508 (PMC12073202; doi:10.3390/nu17091508)
Supplement: Supplementary file 1 [file nutrients-17-01508-s001.zip › nutrients-3578778-supplementary.pdf]

**Table S1** Diagnoses/ Procedures of interest and corresponding ICD-10 codes.

| Diagnosis | ICD 10 codes |
|---|---|
| Non-severe malnutrition<br>(mild to moderate) | E44.0 Moderate protein-calorie malnutrition<br>E44.1 Mild protein-calorie malnutrition<br>E46 Unspecified non-severe protein-calorie malnutrition |
| Severe malnutrition | E40 Kwashiorkor, a severe form of malnutrition characterized by nutritional edema, dyspigmentation of skin and hair<br>E41 Nutritional Marusmus<br>E42 Marasmic kwashiorkor<br>E43 Unspecified severe protein-calorie malnutrition<br>R64 Cachexia (severe wasting of muscle and fat mass, often associated with underlying chronic illnesses) |
| ESRD | N185, N186 |
| Acute kidney injury | N170, N171, N172, N178, N179, N19, N990, O904 |
| Peritoneal dialysis | 3E1M39Z |
| Hemodialysis | 5A1D70Z, 5A1D80Z, 5A1D90Z |
| Kidney transplantation | Z94.0 |
| Diabetes mellitus | E10.0, E10.1, E10.6, E10.8, E10.9,<br>E11.0, E11.1, E11.6, E11.8, E11.9,<br>E12.0, E12.1, E12.6, E12.8, E12.9,<br>E13.0, E13.1, E13.6, E13.8, E13.9,<br>E14.0, E14.1, E14.6, E14.8, E14.9<br>E10.2–E10.5, E10.7, E11.2–E11.5,<br>E11.7, E12.2–E12.5, E12.7, E13.2–<br>E13.5, E13.7, E14.2–E14.5, E14.7 |
| Hypertension | I10.X |
| Coronary artery disease | I2510, I7090, I1779, I519, I259, I700, I672, I652, I25798, I25791, I25709, I25708, I25700, I25701, I2584, I2583, I25119, I25118, I25110, I25111, I25119, I25810, I7209, I25811, I25759, I25750, I25751, I25759, I70209, K511, I78, I7090, I672, G9519, I270 |
| Smoking | Z87891, Z720, Z5301, Z716 |
| Alcohol use | F1012, F1020, F1090, F1022, F1092 |
| Volume overload | E8770, E8771, E8779 |
| Sepsis | A409, A412, A4101, A411, A403, A414, A4151, A4152, A4153, A4159, T80219A, T80211A, T80212A, T8022XA, T8029XA, K6811, N390 |

| | |
|---|---|
| Catheter-related blood stream infection | T80.211A |
| Congestive heart failure | I09.9, I11.0, I13.0, I13.2, I25.5, I42.0, I42.5–I42.9, I43.x, I50.x, P29.0 |
| Peripheral vascular disease | I70.x, I71.x, I73.1, I73.8, I73.9, I77.1, I79.0, I79.2, K55.1, K55.8, K55.9, Z95.8, Z95.9 |
| Cerebrovascular disease | G45.x, G46.x, H34.0, I60.x–I69.x |
| Dementia/cognitive impairment | F00.x–F03.x, F05.1, G30.x, G31.1 |
| Moderate or severe liver disease | I85.0, I85.9, I86.4, I98.2, K70.4, K71.1, K72.1, K72.9, K76.5, K76.6, K76.7 |
| Diabetes without chronic complication | E10.0, E10.1, E10.6, E10.8, E10.9, E11.0, E11.1, E11.6, E11.8, E11.9, E12.0, E12.1, E12.6, E12.8, E12.9, E13.0, E13.1, E13.6, E13.8, E13.9, E14.0, E14.1, E14.6, E14.8, E14.9 |
| Diabetes with chronic complication | E10.2–E10.5, E10.7, E11.2–E11.5, E11.7, E12.2–E12.5, E12.7, E13.2–E13.5, E13.7, E14.2–E14.5, E14.7 |
| Any malignancy, including lymphoma and leukemia, except malignant neoplasm of skin | C00.x–C26.x, C30.x–C34.x, C37.x–C41.x, C43.x, C45.x–C58.x, C60.x–C76.x, C81.x–C85.x, C88.x, C90.x–C97.x |
| Metastatic solid tumor | C77.x–C80.x |
| **Procedure** | **ICD-10 codes** |
| Blood transfusion | 30233N1, 30243N1 |
| Need for vasopressors | 3E033XZ |
| TPN use | 3E0336Z, 3E0436Z |
| Mechanical ventilation | 5A0935Z, 5A0955Z, 5A09357, 5A09358, 5A09359, 5A0935A, 5A0935B, 5A09457, 5A09458, 5A09459, 5A0945A, 5A0945B, 5A09557, 5A09558, 5A09559, 5A0955A, 5A0955B, Z991, Z9910, Z9911, Z9912 |
